# Tissue Engineering Approaches to Uncover Therapeutic Targets for Endothelial Dysfunction in Pathological Microenvironments

**DOI:** 10.3390/ijms23137416

**Published:** 2022-07-03

**Authors:** Dimitris Ntekoumes, Sharon Gerecht

**Affiliations:** 1 Department of Biomedical Engineering, Duke University, Durham, NC 27708, USA; dimitrios.ntekoumes@duke.edu; 2 Department of Chemical and Biomolecular Engineering, Johns Hopkins University, Baltimore, MD 21218, USA

**Keywords:** tissue engineering, endothelial cells, disease modeling, hydrogel, organ-on-chip, 3D printing, vascular grafts, organoids

## Abstract

Endothelial cell dysfunction plays a central role in many pathologies, rendering it crucial to understand the underlying mechanism for potential therapeutics. Tissue engineering offers opportunities for in vitro studies of endothelial dysfunction in pathological mimicry environments. Here, we begin by analyzing hydrogel biomaterials as a platform for understanding the roles of the extracellular matrix and hypoxia in vascular formation. We next examine how three-dimensional bioprinting has been applied to recapitulate healthy and diseased tissue constructs in a highly controllable and patient-specific manner. Similarly, studies have utilized organs-on-a-chip technology to understand endothelial dysfunction’s contribution to pathologies in tissue-specific cellular components under well-controlled physicochemical cues. Finally, we consider studies using the in vitro construction of multicellular blood vessels, termed tissue-engineered blood vessels, and the spontaneous assembly of microvascular networks in organoids to delineate pathological endothelial dysfunction.

## 1. Introduction 

Tissue engineering aims to recreate functional human tissues in vitro to understand tissue development and function and develop therapeutics. A major bottleneck in the field is the efficient vascularization of engineered tissues [1,2,3,4]. Blood vessels are lined with a monolayer of endothelial cells (ECs) that interacts directly with the bloodstream while serving as a barrier between the blood and the adjacent tissue. ECs perceive and respond to changes in their surrounding microenvironments to maintain vascular homeostasis [5]. Dysfunction of the vascular endothelium is a component of numerous pathologies, including cardiovascular disease [6,7,8], diabetes [9,10,11,12,13,14], kidney disease [15,16,17], pathogen invasion [18,19,20], and cancer [21,22,23,24,25]. The typical manifestations of impaired endothelium function include disrupted barrier function, elevated reactive oxygen species (ROS), reduced nitric oxide production, and increased leukocyte adhesion [26,27,28]. Therefore, understanding the molecular basis contributing to endothelium impairment is pivotal for developing therapeutics for various disorders. Complementing simple two-dimensional (2D) cell cultures with in vivo models has provided great insight into the mechanisms governing the assembly of healthy and pathological endothelium. However, animal models often hamper the development of novel therapeutics as they fail to faithfully recapitulate the molecular mechanisms involved in human disease [29,30,31,32]. Therefore, interdisciplinary efforts are focused on developing advanced platforms to bridge the gap between in vitro testing and clinical translation [33]. Tissue mimicry platforms include engineered hydrogels, three-dimensional (3D) printed blood vessels, organs-on-a-chip systems, tissue-engineered blood vessels (TEBVs), and organoids [2]. A common trait of all these advanced platforms is that they provide biophysical and biochemical cues, such as soluble factors, flow, and structural support, thus dictating a more physiologically relevant microenvironment for the ECs than traditional cell cultures. Hydrogels, comprised of crosslinked polymer networks that absorb large amounts of water, provide a versatile approach to mimicking the native architectures of tissues and the extracellular matrix (ECM) [34]. Besides providing structural support to ECs, ECM is involved in numerous signaling pathways that regulate EC migration, proliferation, and vascular morphogenesis [35,36,37,38]. The advent of 3D printing has been a significant step toward the prospect of improving spatiotemporal control over cellular behavior. Yet, using 3D printing techniques to fabricate blood vessels is relatively in its infancy. Research in this area mainly focuses on developing biocompatible and highly tunable materials that serve as improved bioinks and optimizing fabrication techniques to achieve the more distinct topological features of the vasculature [39,40,41,42,43,44,45,46]. Progress in microfabrication techniques has led to the development of organs-on-a-chip technologies that allow a cell culture under flow, paving the way to recapitulate the organ-level pathophysiology [2,33]. Microfluidic devices have been extensively utilized [47] as in vitro platforms to emulate the physiological functions of organs, such as the lung [48,49,50], liver [51,52,53], and heart [54,55,56], as well as to mimic pathological states, including cancer [21,25,57], neurological disorders [57], and pathogen invasions [20,58,59]. Microfluidic platforms have also been utilized to investigate self-assembled miniaturized organs and the vascularization of organoids, with great strides being made in creating vascularized brain [58,60], heart [59], and kidney [61] organoids. Recently, the fabrication of a multiorgan chip [62] in which mature human heart, liver, bone, and skin tissues were linked through circulating a vascular flow revealed clinically relevant pharmacokinetic and pharmacodynamic profiles upon drug administration. In this review, we summarize the recent progress in tissue-engineered platforms that have been used to study the underlying molecular signaling in pathologies associated with dysfunctional endothelium.

## 2. Engineered Hydrogels for Vascular Formations

**Figure 1:**
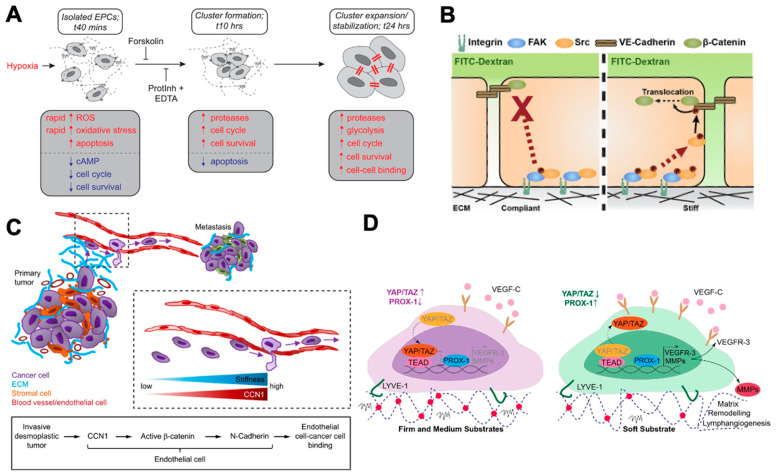
Engineered hydrogels for elucidating the molecular mechanisms of ECs in pathological microenvironments. (**A**). Hypoxia results in rapid ROS accumulation and triggers cAMP downregulation to protect the cells from oxidative stress. Protease upregulation facilitates ECM degradation and initial cluster formation. At later time points, ECFC clusters are stabilized through increased cell–cell adhesion and upregulation of carbohydrate metabolism. The schematic was adapted from [75]. (**B**). ECM stiffness modulates endothelial adherens junctions’ integrity. Activation of FAK on stiff matrices promotes the translocation of phosphorylated Src to the adherens junctions, triggering the dissociation of β-catenin and subsequently disrupting the endothelial barrier. Reprinted/adapted with permission from [90] 2022, John Wiley and Sons. (**C**). Enhanced tumor stiffness upregulates the expression of CCN1 in ECs. CCN1 mediates an N-cadherin expression in a β-catenin-mediated fashion, while the N-cadherin expression promotes the adhesion of cancer cells to the vascular endothelium. The schematic was adapted from [91]. (**D**). YAP/TAZ modulates lymphangiogenesis on different substrate stiffnesses. On stiff substrates, YAP/TAZ binds to the PROX-1 promoter, ultimately inhibiting the transcription of the PROX-1 targets VEGFR-3 and MMPs. LECs cultured on compliant substrates facilitate the cytoplasmic translocation and subsequent degradation of YAP/TAZ. An increased expression of VEGFR-3 and MMPs leads to enhanced ECM remodeling and lymphatic tube formation. Reprinted/adapted with permission from [98] 2022, John Wiley and Sons.

Hydrogels are naturally derived or synthetic polymer materials that can be engineered to mimic the ECM and recapitulate various microenvironmental cues, including stiffness, matrix viscoelasticity, and integrin specificity [63,64]. Therefore, such biomaterial platforms serve as attractive in vitro approaches to delineating the effects of ECM on EC behavior and subsequent vascular network formations [34]. For example, fibronectin-based hydrogels that preferentially bind α3/5β1 or αvβ3 integrins have been utilized to explore integrin specificity as a vascular morphogenic signal. This study demonstrated that only the α3/5β1-specific scaffolds promoted a robust vascular network formation in vitro and in an in vivo ischemic stroke model [65]. Molecular oxygen (O_2_) is central to all multicellular organisms on Earth as it is consumed in many biochemical reactions that regulate cell survival, metabolism, migration, and differentiation [66,67,68]. Cellular adaptation to hypoxia is modulated primarily by stabilizing hypoxia-inducible factors (HIFs) [68]. The accumulation of HIFs promotes angiogenesis during embryonic development, tumor progression, and tissue regeneration due to the upregulation of numerous pro-angiogenic factors, such as the vascular endothelial growth factor (VEGF) and matrix metalloproteinases (MMPs) [69]. In an HIF-independent fashion, the EC hypoxic response is also mediated through the mammalian target of rapamycin signaling [70,71] and the production of ROS [72]. To recapitulate a three-dimensional (3D) hypoxic environment and gradients in vitro, a novel hydrogel platform has been developed, the gelatin-based hypoxia-inducible hydrogel (Gel-HI) [73]. Using this system, it has been shown that the vascular assembly of endothelial colony-forming cells (ECFCs, a subtype of endothelial progenitors) in hypoxia is mediated through the accumulation of HIFs, leading to upregulation in MMPs, thus enabling matrix degradation and subsequent network assembly [73]. Next, using this hydrogel, a novel mechanism for the cluster-based vasculogenesis of ECFCs was proposed [74]. Following exposure to a 3D hypoxic gradient, rapid ROS production, but not HIF stabilization, facilitates ECFC clustering. Simultaneously, MMP-1 is upregulated, thus allowing ECFCs to degrade the surrounding ECM. Vascular endothelial-cadherin (VE-cad), integrin-β2, and intracellular adhesion molecule-1 (ICAM-1) stabilize the ECFC clusters. Further, adding an external crosslinking agent enhances branching and sprouting from the ECFC clusters, thus highlighting the importance of matrix stiffness in vascular network formation [74]. A subsequent study [75] found that generating distinct Gel-HI layers allows for mechanistic studies. RNA sequencing analysis revealed the time-dependent regulation of numerous biological functions that govern the hypoxic-cluster formation, including cell survival, apoptosis, cell cycle progression, and carbohydrate metabolism. The downregulation of cyclic adenosine monophosphate signaling allowed for cell survival in a high-stress microenvironment generated by the rapid upregulation of oxidative stress and ROS (Figure 1A). Further cluster stabilization was facilitated through the upregulation of vascular cell adhesion molecule 1 (VCAM-1) and the carbohydrate metabolism in hypoxic conditions [75]. Therefore, the targeted delivery of Gel-HI with discretized oxygen gradients holds promise as the therapeutic system for eliciting angiogenic responses in ischemic diseases. Vascular stiffening occurs upon aging [76] and accompanies the progression of various pathologies, including hypertension [77], atherosclerosis [78], and myocardial ischemia [79]. Changes in the microarchitecture of the ECM, such as increased collagen deposition and ECM protein crosslinking, contribute to increased matrix stiffness. ECs activate mechano-sensitive signaling pathways in response to shear stress and ECM mechanics [80,81,82,83,84,85]. Platelet endothelial cell adhesion molecule-1 has been shown to dictate EC responses to applied force through the protein kinase A (PKA)/RhoA pathway. The inhibition of the force-induced activation of PKA restores the growth of focal adhesions and the EC response to shear stress in vitro and in vivo. Increased stromal stiffness, a hallmark of cancer progression [86,87], was shown to promote a leaky phenotype in tumor vessels due to enhanced endothelial permeability [88]. Specifically, Bordeleau et al. [88] utilized nonenzymatic glycation reactions for the secondary crosslinking of collagen I hydrogels, emulating the increased ECM stiffness of solid tumor tissue [89]. EC spheroids showed high MMP-14 expressions in the stiff hydrogels, leading to an increased angiogenic sprouting compared to compliant matrices. VE-cad staining revealed that the newly formed vessels had wider cell–cell junctions, suggesting that an increased ECM stiffness disrupts the EC barrier function. In addition, ECs cultured on compliant polyacrylamide substrates exhibited continuous VE-cad- and β-catenin-mediated junctions and decreased the permeability compared to stiff substrates. The increased stromal stiffness was further found to disrupt the barrier function and enhance the permeability of tumor vasculature in vivo [88]. In a subsequent study, the authors proposed that the stiffness-mediated activation of focal adhesion kinase (FAK) promotes the translocation of phosphorylated Src to the EC adherens junctions. VE-cad is subsequently phosphorylated, triggering the dissociation of β-catenin and thus disrupting the EC barrier integrity (Figure 1B) [90]. In a different set of studies, proteome analysis was employed to investigate EC responses in compliant and stiff polyacrylamide/fibronectin matrices, thus mimicking healthy and tumor microenvironments, respectively [91]. The stiffness-induced upregulation of N-cadherin, a protein heavily involved in cancer metastasis [92], was dependent on CCN family member 1 (CCN1) in a β-catenin-mediated manner. The authors further examined the role of CCN1 in cancer–endothelium interactions through the co-culture of ECs and PC3 prostate cancer cells in matrices with varying stiffnesses (Figure 1C). CCN1 knockdowns resulted in decreased adhesion of cancer cells to the EC monolayer in stiff matrices, while in vivo CCN1 knockouts in a highly metastatic mouse melanoma model also revealed decreased cancer cell adhesion to blood vessels and reduced metastasis [91]. The lymphatic system is responsible for numerous biological processes, including lipid transportat, immune cell trafficking, and maintaining interstitial fluid homeostasis [93,94,95,96]. Recent efforts have been focused on engineering the lymphatic vasculature [97,98,99] due to its potential to rescue various disease phenotypes, such as Alzheimer’s, lymphedema, cardiovascular disease, and impaired wound healing [96,98]. Alderfer et al. [98] generated tunable hyaluronic acid (HA)-based hydrogels with a wide range of stiffness to study lymphatic cord formation in vitro. Human lymphatic ECs (LECs) seeded on both stiff and soft HA hydrogels exhibited key lymphatic markers, including lymphatic-vessel endothelial hyaluronan receptor-1 and prospero-related homeobox-1 (PROX1). In LECs cultured on stiff substrates, the Yes-associated protein (YAP)/transcriptional co-activator with a PDZ-binding motif (TAZ) enters the nucleus and binds to the PROX-1 promoter, subsequently suppressing the transcription of PROX-1 as well as its targets VEGF receptor -3 (VEGFR-3) and MMPs. Decreasing matrix stiffness promotes the translocation and subsequent cytoplasmic degradation of YAP/TAZ, resulting in enhanced transcription of PROX-1, MMP-14, and VEGFR-3. The binding of VEGF-C to VEGFR-3 and enhanced matrix remodeling by MMP-14 facilitate lymphatic tube formations in soft matrices (Figure 1D). Besides matrix stiffness, recent approaches to engineering more physiologically relevant hydrogel systems have been focused on better recapitulating the viscoelasticity of the native soft tissues [100,101,102,103,104,105,106,107,108]. The inherent property of a polymeric material to reconstitute in response to external deformation is known as stress relaxation. Viscoelastic hydrogels crosslinked with dynamic covalent bonds or reversible physical interactions exhibit stress relaxation and have been employed to modulate cell behaviors in mesenchymal stem cells [100,101,102,103,104], induced pluripotent stem cells (iPSC) [101], myoblasts [105], neural progenitor cells [108], and cancer cells [106,107]. Wei et al. [109] highlighted stress relaxation as a necessary matrix property that regulates vascular morphogenesis by utilizing gelatin/dextran-based hydrogels crosslinked with dynamic or conventional (nondynamic) covalent bonds. Irrespective of matrix stiffness, the dynamic hydrogels’ stress relaxation facilitated integrin clustering, leading to stable focal adhesions. FAK activation enhanced the remodeling of the ECM through increased MMP expression and ECM depositions, thus promoting vascular morphogenesis in the dynamic hydrogels [109].

## 3. Three-Dimensional (3D) Bioprinted Blood Vessels

**Figure 2:**
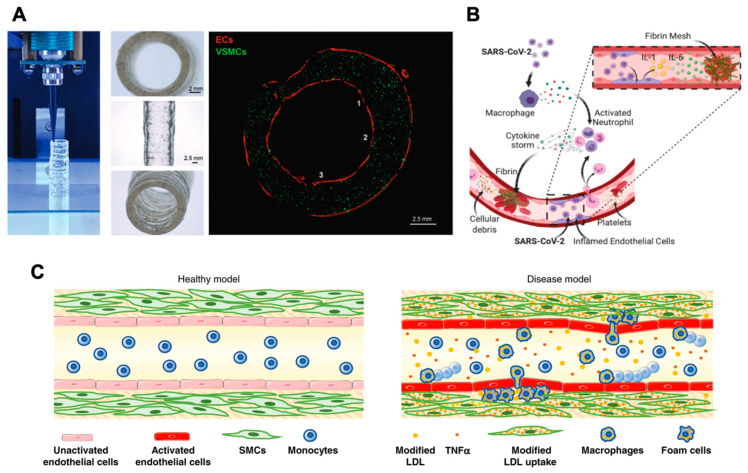
Advanced tissue-engineered approaches to modeling endothelial disease. (**A**). ECs and VSMCs co-cultured in a 3D bioprinted vascular model. Reprinted/adapted with permission from [113]. 2022, John Wiley and Sons. (**B**). Infection with SARS-CoV-2 induces the release of cytokines, including IL-1 and IL-6, that promote platelet activation and initiation of the coagulation cascade. The schematic was adapted with from [145]. (**C**). TEBV model for the onset of atherosclerosis. Treatment with eLDL and TNF-α results in activation of the vascular endothelium and leads to macrophage infiltration and foam cell accumulation on the vessel walls. The schematic was adapted from [155].

The scarcity of organs for transplantation has pushed the field of tissue engineering to seek alternative methods for obtaining readily available, fully functional tissue for implantation [41,110,111,112]. Three-dimensional bioprinting has emerged as a promising technology for recapitulating healthy and diseased tissue constructs in a highly controllable and patient-specific fashion [43,44,46]. Gold et al. [113] generated a 3D printed blood vessel model that, upon cytokine stimulation, mimics thrombo-inflammatory responses observed in vivo. A novel colloidal bioink comprised of gelatin methacrylate (Gel-MA), poly(ethylene glycol) diacrylate (PEGDA), and nanosilicates (nSi), allowed for the fine-tuning of the system’s mechanical properties. Gel-MA/PEGDA/nSi also exhibited shear-thinning behavior, followed by an immediate recovery of its internal structure, thus protecting the cells from shear-induced damage and enabling cell survival for up to seven days. The native architecture of the medial layer was reconstructed by encapsulating vascular smooth muscle cells (VSMCs) in the composite bioink, followed by the printing of a hollow, cylindrical free-standing structure (Figure 2A) [113]. The direct seeding of human umbilical vein ECs (HUVECs) on the 3D-printed construct allowed the formation of an endothelialized luminal structure that mimics the native blood vessel. The authors utilized tumor necrosis factor-α (TNF-α) stimulation and blood exposure to assess whether the 3D bioprinted blood vessel facilitated crosstalk between the EC monolayer and VSMCs. The EC lining on the inner wall prevented clot formations and maintained the printed vessel’s patency. VSMCs conserved the VE-cad junctions while decreasing the expression of inflammatory markers, including IL-8, IL-6, IL-1β, and monocyte chemoattractant protein-1 (MCP-1) compared to EC monocultures, suggesting that VSMC-EC communication promotes a less thrombotic EC phenotype [113]. More recently, a 3D-printed middle cerebral artery (MCA) model was utilized to decipher the mechanism of cerebral EC damage in response to severe acute respiratory syndrome coronavirus-2 (SARS-CoV-2) exposure [114]. Infection with SARS-CoV-2 has been associated with an increased occurrence of cerebrovascular diseases, such as intracerebral hemorrhage and ischemic stroke. However, the underlying mechanism remains elusive [115]. Kaneko et al. [114] fabricated a patient-specific polydimethylsiloxane (PDMS) MCA model based on 3D reconstructed blood vessel images acquired from computed tomography angiographs. The perfusion of the MCA model triggered the expression of human angiotensin-converting-enzyme-2 (hACE2), the receptor for SARS-CoV-2, in human brain microvascular ECs (BMECs) compared to 2D culture. The 3D flow culture further facilitated the binding of the viral spike protein (S protein) to hACE2, an event responsible for the attachment of SARS-CoV-2 and a subsequent cellular entry. Three-dimensional bioprinted models have also been utilized to investigate the pathophysiology of renal reabsorption [116]. Proximal tubule (PT) epithelial cells and glomerular microvascular ECs (GMECs) were embedded in adjacent perfusable channels, while a permeable gelatin-fibrin ECM allowed cell–cell communication. Sodium-glucose co-transporter 2 (SGLT-2), a well-established mediator of glucose reabsorption [117], was upregulated in the presence of GMECs, indicating a positive crosstalk between endothelium- and epithelium-lined conduits. The 3D printed PT model showed increased glucose reabsorption compared to 2D transwell controls. Exposing the 3D printed PT model to hyperglycemic conditions, hallmark of diabetes, resulted in increased cell stress and disruption of the cell–cell junctions of GMECs. Accordingly, administering the SGLT2 inhibitor decreased the reabsorption rate and protected the endothelium against glucose excess [116]. Three-dimensional bioprinting efforts have also been focused on reconstructing the complexity of the tumor microenvironment [118]. Langer et al. [119] generated a 3D bioprinted multicellular tumor model that exhibits whole-tissue level organization. The spatial organization of the tumor was achieved using distinct stromal and cancer bioinks. HUVECs and fibroblasts constitute the stroma, while HUVECs and breast cancer cell lines assemble the cancer compartment. A reversibly crosslinked gelatin-alginate hydrogel facilitated the initial integration of the two bioinks, and it was subsequently removed from the structure, thus allowing for the generation of a scaffold-free 3D bioprinted tumor model. The scaffold-free architecture accommodated close interactions between cancer epithelial cells and stromal fibroblasts and the self-assembly of HUVECs into capillary-like networks. The authors further assessed the efficacy of well-established chemotherapeutics using the 3D printed model, including the VEGF inhibitor sunitinib. The anti-VEGF treatment revealed a striking reduction in the EC networks with increased collagen depositions in 3D bioprinted and in vivo tissues. Furthermore, Chen et al. reconstituted the colorectal cancer (CRC) microenvironment using HCT11 cancer cells, cancer-associated fibroblasts, and tumor-associated endothelial cells (TECs) seeded on a 3D printed polycaprolactone/collagen I scaffold [120]. Several genes associated with the activation of stromal cells were upregulated in the 3D printed CRC model, including tumor endothelial marker 1 (TEM-1) [121], tumor endothelial marker 8 (TEM-8) [122], and tenascin C [123], while increased ECM secretion was also observed. The authors demonstrated that the HCT11 cells are organized into vascular-like cavities, which is indicative of vasculogenic mimicry and associated with poor survival in cancer patients [124,125]. RNA sequencing revealed analogous signaling between the 3D printed model and in vivo tumor compared to 2D control. The authors also showed that the Wnt activation in tumor cells is fibroblast dependent, while the Wnt/β-catenin pathways mediate the pro-angiogenic responses of TECs. The 3D printed CRC model demonstrated higher cell viability for clinically administered CRC drugs, including 5-fluorouracil, cisplatin, and doxorubicin, compared to 2D controls [120]. A recent study [126] exploited the forced overexpression of specific transcription factors (TFs) to simultaneously differentiate human iPSCs into different cell types, regardless of cell culture media compositions. The authors demonstrated that the doxycycline-induced overexpression of ETS translocation variant 2 (ETV2) efficiently differentiates human iPSCs into inducible ECs, while the overexpression of neurogenin-1 (NGN1) rapidly generates inducible neurons from human iPSCs. Both TF-overexpressing cell types preserved their characteristic phenotype when cultured in neural induction medium, whereas WT human iPSCs differentiated into neural stem cells. The authors incorporated ETV2- and NGN1-overexpressing human iPSCs within a gelatin-fibrinogen ECM to generate cell-specific bioinks upon doxycycline treatment. The printed inducible EC filaments exhibited the formation of microvascular networks, while inducible neuron filaments differentiated into neurons that formed neurite networks. Furthermore, the co-printing of WT-, ETV2-overexpressing, and NGN1-overexpressing human iPSCs, followed by doxycycline exposure, gave rise to simultaneous differentiation of neural stem cells, the vascular endothelium, and neurons with a layered tissue architecture that could be maintained for up to 6 days in culture [126].

## 4. Organs-on-a-Chip

Numerous pathological conditions, such as neurological disorders, pathogen invasion, lung inflammation, and thrombotic microangiopathy, are associated with disrupted endothelial function. Most pathologies involve multiple cell types and environmental cues, making it difficult to decouple the effects of a single factor. Microfluidics provide a versatile in vitro platform for recapitulating the endothelial pathophysiology with a high fidelity [33,62,127,128,129,130], thus facilitating the uncovering of the signaling pathways involved in endothelial dysfunction. The neurovascular unit (NVU), composed of vascular cells, glial cells, and neurons, represents the minimal functional unit of the brain [131] and is responsible for maintaining the integrity of the blood–brain barrier (BBB) as well as the regulation of cerebral blood flow [132]. Maoz et al. [133] fabricated a microfluidic model of the NVU comprised of two BBB chips connected on each side of a brain chip to dissect the metabolic crosstalk between the brain vasculature and neurons. Astrocytes and pericytes seeded on top of a transwell plate constitute the perivascular component of the BBB, while confluent human BMECs monolayers make up the vessel compartment. The brain chip consists of human-brain neuronal cells and astrocytes cultured only in the lower compartment. Each BBB compartment is subjected to culture medium flow, allowing components that pass through the BBB to perfuse into the perivascular compartment and diffuse to the brain chip. This novel approach faithfully recapitulates the effects of methamphetamine on the NVU previously reported in vivo and clinical data [133], including the reversible disruption of the BBB function and increased metabolic impact on the perivascular cell metabolism compared to ECs [134,135]. Moreover, for the first time, it was shown that the direct utilization of vascular metabolites in the brain neuronal compartment directly influences the synthesis and secretion of key neurotransmitters, including glutamate and γ-aminobutyric acid [136,137]. The functional human NVU was also established using iPSC-derived BMECs (iBMECs), primary human astrocytes, and pericytes cultured on two parallel microchannels separated by a porous membrane. The presence of astrocytes and pericytes promoted BBB maturation, while upon cytokine perfusion, a leaky tight junction phenotype was observed in the blood compartment. The disruption of tight junctions was followed by retraction of astrocyte protrusions and reduced endfeet-like coverage of the vascular surface, thus indicating functional crosstalk between the brain and the blood compartments. Toward a more personalized approach, the brain compartment was reconstructed using only human iPSC-derived neural cells. The iPSC-derived BBB chip showed similar barrier function to the initial model while maintaining the physiological transendothelial electrical resistance values. The authors further evaluated the potential of the iPSC-derived BBB chip in modeling a severe psychosomatic disease called monocarboxylate transporter 8 (MCT8) deficiency. Compared to controls, the iPSC MCT8-deficient BBB chip showed reduced permeability in T3, a thyroid hormone requiring functional MCT-8. The EC barrier function of the iPSC-based chip was maintained upon a whole human blood perfusion, while TNF-α treatment resulted in blood leakage into the brain compartment [138]. An NVU chip was recently developed to study how *Cryptococcus neoformans*, the most common pathogen causing fungal meningitis, penetrates the BBB [20]. The brain vasculature is reconstructed using a human brain EC-lined channel surrounded by human brain pericytes. The brain compartment of the BBB consists of astrocytes and neurons differentiated from neural stem cells encapsulated in a collagen-HA hydrogel near the brain vasculature. After initially adhering to the BBB, *C. neoformans* formed clusters that were able to penetrate the BBB without substantially disrupting the brain EC tight junctions. *C. neoformans* treatment increased the expression of inflammation-related proteins, including pentraxin 3 (PTX-3), thrombospondin-1 (TSP-1), and IL-8, compared to *C. glabrata* infection, which does not commonly cause fungal meningitis [139]. Screening of factors known to contribute to brain infections but never associated with BBB crossings [140] revealed that Trm7, Dak101, Tlk1, Pka1, and Stb4 promote a BBB penetration by *C. neoformans.* Decellularized organs have emerged as ECM analogs in the field of regenerative medicine. By providing both structural and biochemical cues, decellularized organs can naturally interact with specific tissue cells, thus guiding cell fate and function [141,142,143]. Yuan et al. [144] investigated the role of decellularized whole lung ECM in regulating endothelial phenotypes and function. Following decellularization, rat lungs were mounted in a bioreactor system, while pulmonary microvascular ECs or iPSC-derived ECFCs were suspended in EC culture medium and perfused via the pulmonary artery. Increased barrier function and patent lumen formation suggested the integration of an endothelial lining within the decellularized matrix. Furthermore, repopulation of acellular lungs with ECs resulted in regaining angiogenesis–, cell adhesion–, ECM synthesis-, and cell junction-related markers lost in 2D culture. The authors further investigated the potential of the EC repopulated lungs as a platform for mimicking acute lung injury. Lipopolysaccharide treatment resulted in simultaneous upregulation of pro-inflammatory and matrix remodeling genes, including genes expressing VCAM-1, ICAM-1, and TNF-α, and downregulation of cell junction genes, such as genes expressing tight junction protein 1, occludin (OCLN), and claudin-1 (CLDN-1), in both in vitro and ex vivo native lung controls [144]. SARS-CoV-2-induced endothelial dysfunction has been investigated using human aortic EC-lined microfluidic channels perfused with whole human blood [145]. Satta et al. [145] demonstrated that the viral S-protein (or its D614G mutant) alone enhances fibrin depositions and platelet aggregations. Infection with live SARS-CoV-2 activated the coagulation cascade by promoting the release of endothelial cytokines, including IL-1α, IL-6, and TNF-α, and the expression of endothelial pro-thrombotic markers, such as von Willebrand factor and plasminogen activator inhibitor-1 (Figure 2B) [145]. A treatment with anti-IL-6 antibody or liposome-conjugated hACE2, which competes with S-protein, attenuated blood clot formation. Another recent study [146] utilized endothelialized vasculature-on-a-chip to study the effects of SARS-CoV-2 on the barrier function. HUVECs were cultured on a hollow microfluidic channel composed of a porous polymeric elastomer that allows perfusion. Increased cell death and disruption of the VE-cad junctions were evident 72 h post-SARS-CoV-2 treatments, while pro-inflammatory cytokines increase, including IL-6, IL-8, ICAM-1, and angiopoietin-2 (Ang-2), was further observed. The introduction of peripheral blood mononuclear cells (PMBCs) in the microfluidic device further enhanced the expression of the cytokines above-mentioned compared to a 2D transwell control, while PMBC adhesion disrupted the EC barrier. The presence of a therapeutic Ang-1-derived peptide rescued the virus-induced compromised barrier and reduced cytokine production by attenuating a PMBC adhesion to HUVECs [146]. Qui et al. [147] developed perfusable microvascular-sized channels (about 20 μm) that allow for the long-term study of endothelial barrier dysfunctions and microvascular obstructions occurring in sickle cell disease (SCD) and malaria. An agarose-gelatin hydrogel emulated the tissue stiffness surrounding post-capillary venules and allowed for the formation of a tight endothelial barrier with similar permeability values reported in vivo. TNF-α exposure reversibly increased HUVEC permeability in an E-selectin-, VCAM-1-, and ICAM-1-mediated fashion. Furthermore, free heme, a hemolytic byproduct significantly elevated upon red blood cell (RBC) destructions in sickle cell disease (SCD) and malaria [148], disrupts the EC barrier in a dose-dependent manner. Perfusion of the microvascular channels with RBCs isolated from SCD patients increased EC barrier permeability at the same occlusion site. Increasing the stiffness of the sickle RBCs, a phenomenon associated with a specific sickle RBC subpopulation called irreversibly sickled RBCs, promoted increased endothelial permeability. It was further shown that infections of RBCs with *Plasmodium falciparum*, the parasite that causes malaria, also lead to microvascular occlusions and a twofold increase in the EC barrier permeability, while additional supplementation with TNF-α results in near-complete obstruction of the microchannels. Polacheck et al. [83] fabricated a PDMS-based microfluidic device lined with human dermal microvascular ECs (hMVECs) to elucidate how hemodynamic shear stress regulates endothelial barrier function. Microvessels subjected to flow showed increased expressions of genes associated with Notch signaling, including the gene encoding the NOTCH1 ligand delta-like ligand 4 (DLL4) [149]. The activation of NOTCH1 by shear stress promoted the assembly of the VE-cad-mediated adherens junctions and the redistribution of F-actin to the cortical membrane in a DLL4/γ-secretase manner [83]. Microvessels from hMVECs harboring NOTCH1 and DLL4 knockouts (KO) showed increased barrier permeabilities and disrupted adherens junction integrity, thus highlighting the significance of Notch activation in EC barrier function. For the first time, it was demonstrated that only the transmembrane domain (TMD), but not the intracellular domain (ICD), of NOTCH1 is essential for barrier function and adherens junctions assembly as TMD expression alone suffices to restore adherens junctions in NOTCH1 KO. TMD was also found to directly interact with VE-cad, while robust adherens junctions underflow formation is facilitated by RAC1, a protein that promotes cortical actin bundle formation. The authors proposed that flow-induced NOTCH1 activation promotes VE-cad dissociating from TMD, and TMD is allowed to catalyze the formation of a VE-cad/tyrosine phosphatase LAR/RAC1 guanine-exchange factor TRIO complex, which subsequently induces the activation of RAC1.

## 5. Tissue-Engineered Blood Vessels

Tissue-engineered blood vessels (TEBVs) are typically developed as bypass grafts to treat ischemic heart disease and peripheral vascular disease [150,151]. Ongoing efforts are focused on engineering hierarchical vascular structures that facilitate a better integration with host tissue [152,153,154]. In addition, the ability to cellularize TEBVs with vascular cells (e.g., ECs, SMCs, and fibroblasts) and tissue cells provide a powerful platform for shedding light on the underlying mechanisms of vascular disease, including atherosclerosis and BBB dysfunction. For example, multilayered TEBVs have been utilized to emulate critical features of the onset of atherosclerosis [155]. The adventitial layer was fabricated using a collagen I/human neonatal dermal fibroblast (hNDFs) mixture gelled on top of two interconnected mandrels. A hollow lumen was formed upon mandrel removal, facilitating the perfusion of human smooth muscle cells (hSMCs) and ECFCs. TEBVs exposed to enzyme-modified low-density lipoprotein (eLDL), a key molecule of the atherosclerotic plaque, exhibited reduced vasoactivity and impaired barrier function. Treatment with eLDL and TNF-α led to increased expression of inflammatory markers, including VCAM-1, ICAM-1, E-selectin, and IL-1β. The addition of eLDL, with or without TNF-α, to the perfusion media promoted monocyte accumulation and foam cell formation on the vessel walls (Figure 2C). The eLDL-induced atherogenic phenotype was rescued by pretreatment with lovastatin or the P2Y_11_ inhibitor NF157. The authors fabricated a similar TEBV system comprised of ECFCs and hNDFs to investigate the cellular senescence induced by oxidative stress [156]. TUNEL staining revealed no apoptosis following a 7-day hydrogen peroxide (H_2_O_2_) exposure; however, increased expression of p21 was observed in both ECFCs and hNDFs, indicating enhanced cellular senescence. The H_2_O_2_ treatment further increased VCAM-1 expressions and reduced endothelial-dependent vasodilation of the TEBVs. Atchison et al. [157] fabricated a TEVB model to investigate how Hutchinson–Gilford progeria syndrome (HGPS), a rare genetic condition that causes accelerated aging and death from cardiovascular disease, affects ECs. To this end, iPSC-derived ECs (iECs) and iPSC-derived SMCs (iSMCs) were differentiated from healthy or HGPS iPSC cell lines. Similar to previous work [155,156], a mixture of healthy or HGPS iSMCs and collagen I constituted the medial layer. The intimal layer was established upon perfusion of healthy or HGPS iECs. TEBVs fabricated from HGPS donor cells showed diminished vasoactivity and contractile protein expressions, including myosin heavy chain 1 and α smooth muscle actin, compared to their healthy counterparts. Following perfusion for 1 and 4 weeks, E-selectin and VCAM-1 were highly expressed in HGPS TEBVs, while none of these inflammatory markers was present in healthy donor TEBVs. TEBVs fabricated from HGPS iECs exhibited reduced vasoconstrictive response upon acetylcholine treatment, whether healthy or HGPS iSMCs were used. Additionally, VCAM-1 and E-selectin were upregulated in the TEBVs fabricated from HGPS iECs and healthy iSMCs compared to TEBVs fashioned from HGPS iSMCs and healthy iECs, suggesting a significant role for the endothelium in HGPS. To study brain endothelial behavior, Linville et al. [158] fabricated a tissue-engineered brain microvessel (TEBMV) model seeded with iPSC-derived human BMECs (iBMECs). Brain microvessel channels patterned in genipin-crosslinked collagen I were lined with iBMECs. BBB microvessels were positive for typical tight junction markers, including zona occludens-1 (ZO1), claudin-5 (CLDN-5), and OCLN, and displayed robust physiological barrier function. Treatment with mannitol, a clinically used molecule for BBB openings that improves the delivery of therapeutics to the brain [159], yielded a 15-fold increase in permeability, thus confirming a BBB opening. TNF-α exposure of the iBMECs resulted in upregulated expression of ICAM-1 and VCAM-1 and increased adhesion of human peripheral-blood mononuclear monocytes without altering the barrier permeability. This model was subsequently used to study the reversible BBB opening mediated by melittin, a membrane-active peptide [160]. Melittin induced reversible BBB opening in a dose-dependent manner, with higher doses resulting in increased paracellular permeability and localized microvessel apoptosis, which led to focal leaks.

Further examination revealed that iBMEC-exposed melittin exhibited increased swelling and disrupted ZO1-mediated tight junctions. Melittin also induced reversible opening of the BBB in the mouse microvasculature without neurological damage, suggesting that melittin could potentially be harnessed for targeted drug deliveries into the brain. More recently, Chung et al. [161] utilized the same TEBMV to model the effects of acute and chronic oxidative stress on the BBB, a shared pathology in neurodegenerative disorders [162,163,164]. Acute or chronic exposure to H_2_O_2_ resulted in enhanced barrier permeability characterized by focal leaks, local loss of adhesion (delaminations), or both. In both types of exposures, H_2_O_2_-induced damage was resolved by the re-adherence of the endothelium to the ECM and reversal of dye leakage. Bulk RNA sequencing revealed that cell cycle dysfunction-associated genes, such as *CDKN1A*, *GADD45A*, and *HSPA1B*, were more prevalent under acute oxidative stress conditions. At the same time, chronic exposure resulted in the upregulation of immune cell chemotaxis and migration-associated genes, including *CCL4*, *CCL20*, and *CXCL1*. Furthermore, the authors demonstrated increased monocyte adhesion four days after acute and chronic H_2_O_2_ exposures.

## 6. Organoids

Advances in understanding stem cell behavior have led to the generation of 3D miniaturized organ systems called organoids [165]. Organoids are typically derived from PSCs that, upon exposure to tissue-specific microenvironmental cues, self-assemble into a 3D structure that resembles the native architecture of the corresponding organ [166]. Organoids are promising platforms for modeling developmental and genetic disorders by utilizing patient-derived iPSCs or introducing disease-causing mutations [166,167]. Since the development of the first stem cell-derived 3D crypt–villus organoid [168], numerous efforts have been made to generate organoids specific to the gut [29,168,169,170,171], kidney [64,172,173], brain [172,174,175], stomach [173,176,177], and pancreas [178,179,180], among others [166]. Nevertheless, due to a lack of continuous oxygen and nutrient supplies, these organoids cannot be sustained for long periods, indicating that ECs are essential for organ development [181,182]. In a seminal study, Wimmer et al. [14] generated a human PSC-derived blood vessel organoid model to elucidate how EC dysfunction leads to diabetic vasculopathy. Vascular organoids exhibited 3D mature vascular networks with tightly associated ECs and pericytes, allowing them to grow in mice for up to 6 months. Hyperglycemic exposures and treatments with pro-inflammatory cytokines recapitulate the diabetic microenvironment. Treating the vascular organoids with high glucose levels and TNF-α/IL-6 resulted in increased ECM protein deposition, including collagen IV, fibronectin, laminin, and perlecan. In addition, the organoids exhibited thickening of the basement membrane layer, consistent with observations in type 2 diabetes patients (T2D). Blockade of the γ-secretase pathway with DAPT, but not treatment with anti-diabetic drugs, abolished collagen IV overexpression in the periphery of the blood vessels and mitigated the thickening of the basement membranes of vascular organoids transplanted in diabetic mice. The authors further identified the γ-secretase target NOTCH3 and its ligand DLL4 as the specific targets of DAPT. NOTCH3 expressions were mainly observed in pericytes in both diabetic and nondiabetic organoids transplanted in mice and in both T2D patients and healthy individuals. Furthermore, the expression of HES5, a NOTCH3 downstream target, was more pronounced in the pericytes of both transplanted diabetic organoids and T2D patients than in their healthy counterparts. Inhibition of NOTCH3 attenuated the thickening of the basement membranes in diabetic vascular organoids transplanted in mice. In a recent study [183], iPSC-derived blood vessel organoids were utilized to study the effects of recombinant soluble ACE2 (hrsACE2) on SARS-CoV-2 growth. SARS-CoV-2 is located primarily in the lungs; however, the virus’s size suggests that it must first infect the blood vessels to reach other tissues. SARS-CoV-2 RNA was detected in the organoids 3 days post-infection, and the viral load significantly increased after 6 days, indicating an active SARS-CoV-2 replication. Treatment of blood vessel organoids with hrsACE2 reduced the infection, demonstrating that hrsACE2 can inhibit the early entry of SARS-CoV-2 in host cells.

## 7. Discussion

Outstanding progress has been achieved over the past few decades in generating in vitro models that emulate endothelial physiology and pathophysiology. Combining novel chemistries with state-of-the-art fabrication techniques has facilitated the incorporation of microenvironmental cues that govern cellular behavior in healthy and diseased vascular endothelia (Table 1). Yet, great strides need to be made toward the precise engineering of human vasculature in vitro. Most tissue-engineered approaches lack standardized manufacturing and operating procedures. The increased complexity of certain biological phenomena necessitates a significant investment of time and resources to set up and customize the in vitro model [184]. Streamlining the production of tissue-engineered platforms, such as organs-on-a-chip, on an industrial scale holds promise for transforming current biological research, allowing nonexpert users to study the biological question at hand without requiring vigorous effort to establish the advanced in vitro system. The use of drug-inert organs-on-a-chip materials would also facilitate this endeavor. Despite being easy to process, PDMS, the most used polymer in organs-on-a-chip, may hinder drug studies due to the intrinsic characteristic of absorbing hydrophobic compounds [33,130]. Recent studies have attempted to tackle this challenge by applying inert coatings or accounting for PDMS drug absorption [185]. Another challenge, more prominent in TEBVs, is proper cell seeding and distribution on an engineered scaffold. Static approaches to cellularizing TEBVs remain largely stochastic as they rely on gravity-mediated cell sedimentation on the TEBV surface and a subsequent cell penetration into the construct [4]. Dynamic seeding techniques involving rotational or centrifugal systems facilitate a more homogeneous cell deposition on the scaffold; however, they can be rather slow (~24 h), thus increasing the risk of contamination or having adverse effects on cell morphology due to high speeds [186]. Top-down approaches, including 3D printing, laser degradation, and layer-by-layer fabrication [4], have emerged as attractive technologies for fabricating tissue-like constructs in a highly controllable fashion. Low cost, simplicity, and flexibility are unique assets of injekt and extrusion bioprinting. However, bioprinting-induced shear stress may cause cell damage and death, while the confinement of nozzles typically limits the printing resolution. Furthermore, structural integrity may be disrupted at the interfaces of droplets and lines in the cases of injekt and extrusion printing, respectively [187]. Recapitulating the vasculature’s dimensional and cellular hierarchy is one aspect that requires further attention [2]. For instance, the reconstruction of human arteries in vitro is typically limited to fabricating a single perfusable patent channel, typically >0.1 mm in diameter, lined with an EC monolayer. Despite obtaining physiological dimensions, the single-layered endothelium is an attribute of the capillaries, where gas exchange occurs, rather than an attribute of larger vessels, such as arteries. Failing to encompass mural cells, such as SMCs, negates the molecular crosstalk between the different cell types, ultimately leading to impaired blood vessel function. Moreover, mural cells in large vessels impact the EC response to flow-derived mechanical cues [188]. In the context of 3D vascular networks fabricated in hydrogels, the absence of mural cells typically leads to the disintegration of an assembled vasculature within a few days. For instance, incorporating pericytes in the hydrogel design would potentially enhance the stability of the vascular networks [182,189]. Excessive ECM remodeling, occurring especially in naturally derived matrices, such as collagen and gelatin, is another factor limiting the study of the engineered vasculature within a narrow time frame [109]. To achieve a higher degree of tunability regarding matrix mechanics, degradability, and integrin binding, the use of dextran [190], poly (ethylene glycol) [191], or HA [192] could also be considered. Organoids, an emerging area that promises to mimic the whole-organ physiology in vitro, may lack adequate oxygen and nutrient supply, leading to growth arrest and limited maturation [2,193]. Incorporating functional vasculature in the organoid design would allow culture for longer periods, potentially permitting the scale-up to centimeter sizes. In addition, current organoid systems are highly heterogeneous, which can be partially attributed to the inherent stochasticity of cell differentiation and the systems’ heavy dependence on Matrigel, a laminin-rich yet largely undefined basement membrane. Matrigel is an important component of organoid cultures, providing structural support and supplementing essential signaling cues that direct organoid formation. Even though great progress has been achieved in reducing the stochasticity that governs organoid formations through well-defined engineered matrices [194], synthetic scaffolds remain primitive compared to Matrigel in guiding cell-driven ECM remodeling [195]. Linking organoids from various tissues, such as the heart, brain, kidney, and liver, with vascular flow, would facilitate the analysis of pharmacokinetics/pharmacodynamics at a whole-organism level in vitro. Higher fidelity of the engineered in vitro models can be attained by developing tissue-specific vasculature, thus emulating in vivo phenotype and functionality. Although HUVECs are considered the “gold standard” approach to studying the vascular endothelium, they do not encompass the entirety of organ-specific EC responses. Progress in differentiation protocols to obtain tissue-specific ECs has promising clinical prospects [182]. The use of patient-derived ECs to investigate genetic diseases, identify drug targets, and unravel disease mechanisms has already been examined [157,196]. Advances in transcriptome analysis and further functional comparisons with mature tissue counterparts could potentially assist in the discovery of novel disease- or organ-specific EC markers [182]. Endothelial metabolism is another aspect often overlooked when studying disease mechanisms [28]. A recent study [133] examining the effects of methamphetamine on a tissue-engineered model of the NVU identified a previously unknown interplay between the BBB and neurons, suggesting a potential metabolic role for the brain microvasculature in the progression of neurological diseases. Furthermore, the ketogenic diet was shown to stimulate lymphatic vessel formation in a mouse model of tail lymphedema, leading to a clinical trial examining whether the ketogenic diet elicits similar responses in lymphedema patients [28,197]. Advanced in vitro platforms can decouple the individual effects of the involved components when examining the mechanisms of endothelial pathologies, and therefore, it would be of great benefit to scrutinizing the metabolic aspects of the disease under consideration.

## Figures and Tables

**Table 1:** Tissue-engineered models for the mechanistic study of the vascular endothelium.

Tissue-Engineering Approach	Pathological Endothelial State	Molecular Regulators of Endothelial Response	Setup Complexity
Hydrogels	1. Hypoxia [74,75] 2. Tumor stiffness [90,91]	1. ROS, MMPs, integrin β2, ICAM-1, cAMP, VCAM-1 2. FAK/β-catenin, CCN1/β-catenin, N-cadherin	Medium
3D bioprinted blood vessels	1. Thrombo-inflammation [113] 2. SARS-CoV-2 infection [114] 3. Hyperglycemia [116] 4. Colorectal cancer [120]	1. IL-8, IL-6, IL-1β, MCP-1 2. S-protein 3. SGLT-2 4. TEM-1, T IL-1α, IL-6, EM-8, tenascin-C, Wnt/β-catenin	Medium to high
Organs-on-a-chip	1. Meth exposure [133] 2. MCT8 deficiency [138] 3. *C. neoformans* invasion [20] 4. SARS-CoV-2 infection [145]	1. Glutamate, γ-aminobutyric acid 2. T3 3. PTX-3, TSP-1, IL-8 4. S-protein, IL-1α, IL-6, IL-8, ICAM-1, Ang-2	Medium to high
TEBVs	1. Atherosclerosis [155] 2. Oxidative stress [156,161] 3. HGPS [157]	1. eLDL, VCAM-1, ICAM-1, E-selectin, IL-1β 2. VCAM-1, *CCL4*, *CCL20*, *CXC1* 3. E-selectin, VCAM-1	Medium
Organoids	1. Diabetic vasculopathy [14] 2. SARS-CoV-2 infection [183]	1. NOTCH3, DLL4, HES5 2. hrsACE2	High
